# Dapagliflozin Mediates Plin5/PPARα Signaling Axis to Attenuate Cardiac Hypertrophy

**DOI:** 10.3389/fphar.2021.730623

**Published:** 2021-09-23

**Authors:** Jing Yu, Huanhuan Zhao, Xin Qi, Liping Wei, Zihao Li, Chunpeng Li, Xiaoying Zhang, Hao Wu

**Affiliations:** ^1^ Department of Physiology and Pathophysiology, Tianjin Medical University, Tianjin, China; ^2^ Department of Cardiology, Tianjin Union Medical Center, Nankai University Affiliated Hospital, Tianjin, China; ^3^ Nankai University School of Medicine, Tianjin, China

**Keywords:** dapagliflozin, cardiac hypertrophy, AngII, Plin5/PPARα signaling, mice

## Abstract

**Objective:** The purpose of this study was to investigate the effect of dapagliflozin (DAPA), a sodium-glucose cotransporter 2 inhibitor, on relieving cardiac hypertrophy and its potential molecular mechanism.

**Methods:** Cardiac hypertrophy induced by abdominal aortic constriction (AAC) in mice, dapagliflozin were administered in the drinking water at a dose of 25 mg/kg/d for 12 weeks was observed. Echocardiography was used to detect the changes of cardiac function, including LVEF, LVFS, LVEDd, LVEDs, HR and LV mass. Histological morphological changes were evaluated by Masson trichrome staining and wheat germ agglutinin (WGA) staining. The enrichment of differential genes and signal pathways after treatment was analyzed by gene microarray cardiomyocyte hypertrophy was induced by AngII (2 μM) and the protective effect of dapagliflozin (1 μM) was observed *in vitro*. The morphological changes of myocardial cells were detected by cTnI immunofluorescence staining. ELISA and qRT-PCR assays were performed to detect the expressions levels of cardiac hypertrophy related molecules.

**Results:** After 12 weeks of treatment, DAPA significantly ameliorated cardiac function and inhibited cardiac hypertrophy in AAC-induced mice. *In vitro*, DAPA significantly inhibited abnormal hypertrophy in AngII-induced cardiacmyocytes. Both *in vivo* and *in vitro* experiments have confirmed that DAPA could mediate the Plin5/PPARα signaling axis to play a protective role in inhibiting cardiac hypertrophy.

**Conclusion:** Dapagliflozin activated the Plin5/PPARα signaling axis and exerts a protective effect against cardiac hypertrophy.

## Introduction

Cardiovascular diseases, especially acute myocardial infarction and atherosclerosis, remain the leading cause of death worldwide. In China, the prevalence of cardiovascular disease is rising, causing nearly 4 million deaths in 2016 alone (Liu et al., 2019). Epidemiological studies showed the incidence of cardiac hypertrophy is high worldwide, with the prevalence of about 1/500 (Wasserstrum et al., 2019). Cardiac hypertrophy is the basis of abnormal ventricular remodeling and plays a vital role in the development of most cardiovascular diseases (Pichler et al., 2020). To some extent, elucidating the pathological mechanism of cardiac hypertrophy provides theoretical support for targeted treatment strategies for cardiovascular diseases. Thus, it is urgent to explore the molecular mechanisms underlying cardiac hypertrophy and to seek the effective strategies. Pathological cardiac hypertrophy that occurs in cardiovascular disease is often accompanied by inflammation and myocardial fibrosis, ultimately leading to heart failure (Tham et al., 2015). In addition, the typical pathological basis of cardiac hypertrophy includes the accumulation and deposition of excessive extracellular matrix (ECM) and abnormal cardiomyocyte hypertrophy. At present, the drugs used in clinical treatment of cardiac hypertrophy mainly include angiotensin-converting enzyme inhibitors (e.g., Captopril) (Zhang et al., 2019), angiotensin II receptor blockers (e.g., Losartan) (Gonzalez et al., 2021) and calcium channel blocker (e.g., Amlodipine) (Lu et al., 2016).

As a novel class of hypoglycemic agent, sodium-glucose co-transporter 2 (SGLT-2) inhibitors (e.g., empagliflozin, dapagliflozin) prevented the reabsorption of glucose and sodium from the proximal convoluted tubules, resulting in glycourine and sodium-rich properties. Dapagliflozin increased the amount of glucose excreted in the urine and improved fasting and postprandial blood glucose levels in patients with T2D (Dhillon, 2019). In addition, it has been reported that dapagliflozin has been widely used in the clinical treatment of diabetes and cardiovascular disease. Empagliflozin (EMPA) has been reported to improve cardiac remodeling and cardiac metabolism and ATP homeostasis in rats with myocardial infarction (Yurista et al., 2019). Excitingly, Ye et al. (2017) found that DAPA has a potential cardiovascular protective effect in the treatment of diabetes. Numerous studies (Aimo et al., 2020; Zannad et al., 2020) have shown the importance of DAPA in reducing hospitalization for heart failure in patients with HFrEF and suggest that DAPA may also reduce the risk of cardiovascular events and cardiovascular mortality associated with diabetes. Besides, Lahnwong et al. (2020) reported that acute administration of DAPA could play a protective role by reducing myocardial infarction size and improving left ventricular function in myocardial ischemia/reperfusion rats. It was reported (Zhang et al., 2021) that DAPA ameliorated Ang II-induced cardiac remodeling by regulating TGF-β1/Smad signaling pathway. Despite the potential cardioprotective effects of DAPA has been widely proven, its mechanisms against cardiac hypertrophy remain unclear.

Modern pharmacology reported (Sharma et al., 2004; Chen et al., 2015) that with the development of the pathological process of cardiac hypertrophy, cardiac energy metabolism is disturbed, fatty acid utilization is limited, and the accumulation of lipids will aggravate the occurrence of cardiac hypertrophy and heart failure. Recent studies (Martin and Parton, 2006; Wang et al., 2015) have shown that as dynamic lipid storage organelles, lipid droplets (LDs) is regarded as a key regulator of lipid metabolism in various kinds of cells including adipocytes and cardiomyocytes. The perilipin family of proteins is a classic LDs related protein, among which perilipin 5 (Plin5) is highly expressed in heart, liver and other tissues (Wolins et al., 2006). Wang et al. (2015) confirmed that Plin5 deficiency increased liver lipid metabolism, promoted mitochondrial proliferation, and led to ROS burst. Plin5 deficiency also increased the oxidation of fatty acids in the heart, leading to cardiac dysfunction. Besides, Plin5 deficiency has been reported to exacerbate TAC-induced cardiac hypertrophy and heart failure. Peroxisome proliferator-activated receptor-α (PPARα) is a ligand-activated transcription factor, a member of the nuclear hormone receptor superfamily, which is known for its critical role in the transcriptional regulation of lipid metabolism (Smeets et al., 2008). Peroxisome proliferator-activated receptors (PPARs) are members of the ligand-activated nuclear receptor superfamily and are the key transcriptional regulators that control the capacity for myocardial mitochondrial fatty acid oxidation (FAO) (Stanley et al., 2005). PPARs consists of three member subfamilies: PPAR-α, PPAR-β and PPAR-γ (Montaigne et al., 2021). PPAR-α is highly expressed in cardiomyocytes, and it regulates the expression of key components in fatty acid uptake, esterification, and oxidation through transcriptional activation of genes encoding key proteins in the signaling pathway, and maintains metabolic homeostasis in cardiomyocytes (Kar and Bandyopadhyay, 2018). Loss of PPAR-α has been shown to lead to more pronounced hypertrophic growth and cardiac dysfunction, suggesting a key regulatory role for PPAR-α in cardiac hypertrophy (Smeets et al., 2008). PPARα activator attenuated cardiac hypertrophy by negatively regulating the binding activity of activated protein-1 (AP-1) (Jia et al., 2014; Shimojo et al., 2014) confirmed that Fenofibrate protected against cardiac hypertrophy by activating the PPAR-α signaling and ameliorating myocardial energy metabolism.

Considering the molecular mechanism of Plin5 and PPARα in cardiac hypertrophy, we hypothesized that DAPA might mediate the protective effect of Plin5/PPARα signaling axis. Therefore, we performed the present study to confirm the effect of DAPA towards cardiac hypertrophy in AAC-induced mice and to explore the regulatory effect on Plin5/PPARα signal axis. Our data revealed that the molecular mechanisms by which DAPA attenuated cardiac hypertrophy, which is of great significance for clinical use.

## Methods

### Animals and Groups

Eight-week-old male C57BL/6 mice (23 ± 2 g) were obtained from Beijing Vital River Laboratory Animal Technology Co., Ltd. The mice were kept in wages and given free access to food and water. Mice were housed under suitable conditions, including 12-h light/dark cycle, temperature at 25 ± 2°C and relative humidity 50–60%. Follow-up experiments were started after 1 week of adaptive feeding. Twenty-four mice were randomly divided into three groups: sham group (*n* = 8), AAC group (*n* = 8) and dapagliflozin treatment group (*n* = 8). Sham group and AAC group were treated with saline for 12 weeks. In the treatment group, after the establishment of AAC model, dapagliflozin was administered in the drinking water at a dose of 25 mg/kg/d for 12 weeks (Kraakman et al., 2017). All the animal testing procedures covered in this study complied with the guidelines of the Care and Use of Laboratory Animals and were approved by the Laboratory Animal Center of Tianjin Medical University. All procedures and care methods minimize the suffering of the mice.

### Abdominal Aortic Constriction Model

AAC mice were performed as previously described. (Duan et al., 2007). Briefly, after anesthesia with 2% isoflurane, the mice were placed in the supine position on the platform and cut along the midline of the abdomen to expose the abdominal cavity. Subsequently, the abdominal aorta was ligated with 7-0 silk sutures with a 27G needle. The degree of AAC was controlled by needle removal under suture to ensure the stability and uniformity of the operation. Close the abdominal cavity, carefully suture the skin, and clean the wound. In the sham operation, the silk was removed without ligation. After the operation, the mice were injected with penicillin and put back into the cage when they woke up.

### Echocardiography

After 12 weeks of continuous administration, the mice were anesthetized with 2% isoflurane, and cardiac function was detected by echocardiography. As previously reported (Chai et al., 2020), Doppler images of the lateral and septal mitral annulus were recorded. Heart rate (HR) was recorded by synchronized electrocardiography. Left ventricular ejection fraction (LVEF), fraction shortening (LVFS), left ventricular end-diastolic volume (LVEDV), left ventricular end-systolic volume (LVESV) were calculated.

### Microarray Profiling

After 12 weeks of treatment, the total RNA was isolated from the heart tissue using Trizol reagent (Invitrogen, CA, United States). Determine the concentration of total RNA by fluorescence-based quantitation using an RNA RiboGreen^®^ dye assay (e.g., Quant-iT™ RiboGreen^®^ RNA Reagent and Kit) and the NanoDrop Fluorospectrometer for initial RNA concentration of 5 pg/μL to 1 ng/μL (www.nanodrop.com). RNA integrity was tested using the Agilent 2,100 Bioanalyzer with an RNA LabChip Kit. Global transcriptome analysis of total RNA (50 ng/μl) was performed using the Affymetrix GeneChip Primeview™, according to the manufacturer’s instructions. Hybridization, staining, normalization and data analysis were performed following the standard protocol established by Agilent Technologies, Inc.

### Morphological and Histological Analysis

The mice were anesthetized, the hearts were immediately removed, washed with PBS, and fixed with 4% paraformaldehyde for 24 h. Then paraffin-embedded sections with a thickness of 3–5 μm were stained according to Masson trichrome staining, and wheat germ agglutinin (WGA) staining (Zhu et al., 2018). Masson trichrome staining was performed to evaluate cardiac fibrosis. WGA staining was used to assess the cell size. For WGA staining, briefly, the slides were stained with WGA-FITC labeled antibodies for 30 min at room temperature. The nuclei were stained with DAPI for 5 min. For each analysis, six fields were randomly selected for each sample under the microscope. All histomorphological analyses were performed by pathologists who were unaware of the experimental grouping design.

### Cell Culture and Treatment

Neonatal mouse cardiac myocytes (NMCMs) were isolated from hearts of 1–3 days-old C57BL/6 mice according to the previous report, then the heart was cut into small pieces (Bei et al., 2019). Briefly, the heart was immediately removed and washed with ice-cold PBS. Next the cardiac myocytes were cultured in DMEM (Dulbecco’s modified Eagle’s Medium, Gibco) containing 10% FBS (fetal bovine serum). Then, NMCMs were inoculated in two 25 cm^2^ culture flasks and incubated in 5% CO_2_ for 1 h to isolate cardiomyocytes and cardiomyocytes. The purified mouse primary cardiomyocytes (NMCMs) were cultured for 3 days, which was used for the subsequent experiment. The cells were divided into three groups: control group, AngII group and dapagliflozin group. After the starvation for 4 h, the control group was given a normal medium for 48 h. The cells in AngII group were treated with AngII (2 μM) for 48 h, the medium was changed every 24 h. In the dapagliflozin group, NMCMs were treated with AngII (2 μM) and dapagliflozin (1 μM) for 48 h, the medium was changed every 24 h (Uthman et al., 2018).

### RNA Interference and Groups

After 24 h of cell culture, the purified cardiomyocytes were replaced with the fresh complete medium containing a cardiomyocyte growth supplement. After 24 h, the NMCMs were washed with PBS, and cells were transfected with 1 μM of Lipofectamine 2000 (Invitrogen, CA, United States) and siRNA (si-NC, si-negative control or si-Plin5, Guangzhou RiboBioCo., Ltd., China) in Opti-MEM^®^ I Reduced-Serum Medium according to the manufacturer instructions. 6 h later, cells were treated with AngII (2 μM) and dapagliflozin (1 μM), DMSO or GW6471 (10 μM) for 24 h. The cells were then washed with PBS, harvested, lysed with TRIzol and stored in an −80°C refrigerator for subsequent protein and RNA detection. This part of the experiment is divided into the following groups:

A: AngII + DAPA (dapagliflozin)
B: AngII + DAPA (dapagliflozin) + DMSO
C: AngII + DAPA (dapagliflozin) + DMSO + GW6471 (PPARα inhibitor)
D: AngII + DAPA (dapagliflozin) + si-NC
E: AngII + DAPA (dapagliflozin) + si-Plin5

### Immunofluorescence Staining

Cultured NMCM cells with a density of 1 × 10^5^/mL were harvested and allowed to air dry naturally on the slides. Then the cells were washed with PBS three times and fixed for 15 min with 4% paraformaldehyde at room temperature. 2% bovine serum albumin (BSA) was added to block nonspecific binding at room temperature for 2 h. Then, cTnI antibody was incubated at 37°C for 3 h. After PBS washing, the cells were incubated with biotinylated goat anti-mouse IgG as the secondary antibody, incubated at 37°C for 1 h. Subsequently, the slides were washed with PBS for three times. And the nucleus was counterstained with DAPI. Finally, the cells were observed and photographed under a fluorescence microscope (Olympus, Tokyo, Japan).

### Western Blotting Assay

Heart tissue or NMCMs were lysed in RIPA buffer with PMSF, protease and phosphatase inhibitors. Protein samples were isolated after centrifugation, and the protein concentration was determined by the BCA kit. Protein samples were diluted to 10 μg/μL by PBS, and then stored in −80°C refrigerator for later use. The equal amount of protein was separated in 10% SDS-PAGE gel, transferred to PVDF membranes and blocked with 5% BSA. PVDF membranes were incubated with primary antibodies (STAT1, ab109461; PPARα, ab126285; HMGCS2, ab137043; PDK4, ab214938; GAPDH, ab181602, Abcam, United Kingdom; Plin5, A20418, Abclonal, Wuhan, China) at 4°C overnight. After washing 3 times with TBST and incubated with secondary antibodies at room temperature for 2 h. The bands were visualized with enhanced chemiluminescence (ECL kit) in dark. Then image J software was used to measure the gray value of the target protein bands. GAPDH was used as the loading control. This section of the experiment was repeated independently at least three times.

### Quantitative Real-Time Polymerase Chain Reaction Analysis

Total RNA was isolated from NMCMs and cardiac tissue using Trizol according to standard protocols. The RNA was then determined for purity and concerntration, and cDNAs were synthesized with 500 ng RNA using a reverse transcription kit. qRT-PCR analysis was performed using Takara SYBR Premix Ex Taq™ on Roche LightCycler480 PCR System. The mRNA expression was calculated by −2^ΔΔCT^ method, and GAPDH was used as the loading control.

### Enzyme-Linked Immunosorbent Assay

The contents of ANP, BNP, β-MHC and cTnI in the serum were detected by ELISA kits (Zhang et al., 2019). The test was conducted according to the manufacturers’ instructions.

### Statistical Analysis

The data in the study are presented as the means ± SD, and the data was analyzed using one-way ANOVA followed by the Student *t* test for multiple comparisons by SPSS 22.0 software. The *p* value of less than 0.05 was considered statistically significant.

## Results

### Protective Effects of Dapagliflozin on AAC-Induced Cardiac Hypertrophy in Mice

**FIGURE 1:**
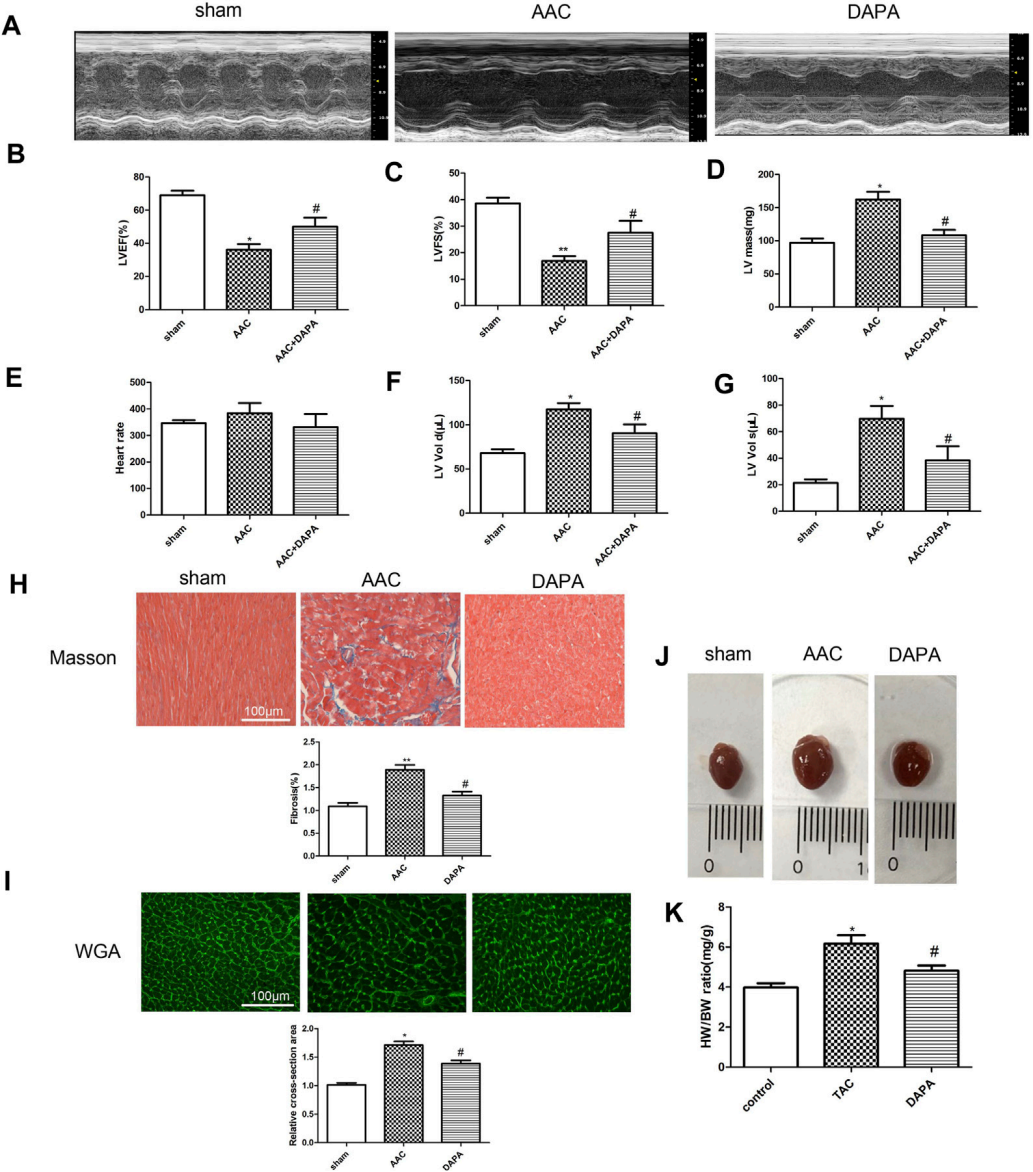
Dapagliflozin alleviated cardiac remodeling and improved cardiac function in AAC-induced mice **(A)** Representative M-mode echocardiography images. In AAC-induced mice, dapagliflozin improved ejection fractions (EF) **(B)** and fractional shortening (FS) **(C)**, LV mass **(D)**, Heart rate **(E)**, LV end-diastolic volume, LVEDV **(F)**, LV end-systolic volume, LVESV, *n* = 8 **(G)**. Images of Masson-stained **(H)** and WGA-stained sections **(I)** in different groups, *n* = 3, original magnification, 400× **(J)** Images of whole heart from mice in different groups after 28 days. **(K)** The heart weight to body weight ratio was determined, *n* = 8. The data were expressed as mean ± SD, *p < 0.05, vs. sham group; #*p* < 0.05, vs. AAC group.

After 12 weeks of treatment with dapagliflozin, the protective effect of dapagliflozin on AAC-induced cardiac hypertrophy was observed. As shown in Figures 1A–C, we found compared with sham group, the left ventricular ejection fraction (LVEF) and fraction shortening (LVFS) in AAC model group were significantly inhibited, indicating a significant decrease in cardiac function in AAC-induced mice. After dapagliflozin treatment, the LVEF and LVFS in the treatment group were improved significantly compared with AAC group. Furthermore, LV mass in DAPA group was reduced markedly compared with AAC group (Figure 1D). According to the echocardiography results (Figures 1E–G), we also obtained HR, LVEDV and LVESV in different groups. There was no significant difference in HR between AAC group and treatment group. While, LVEDV and LVESV in AAC group were significantly increased compared with those in sham group, which was consistent with previous reports of cardiac hypertrophy (Ye et al., 2020). After treatment, LVEDV and LVESV were decreased significantly compared with AAC group. The results suggested that dapagliflozin can significantly improve cardiac function in AAC-induced mice. Masson staining results (Figure 1H) showed that dapagliflozin significantly blunted the myocardial fibrosis. WGA staining results (Figure 1I) were used to detect the changes of cell size. Consistent with the H&E results, dapagliflozin significantly inhibited abnormal hypertrophy of cardiomyocytes after treatment. We observed the changes of the heart as a whole (Figures 1J,K), and we found that the abnormal enlargement of the heart induced by AAC was significantly improved in the treatment group. The ratio of HW to BW was increased in AAC-induced mice, which could be reversed by dapagliflozin. Taken together, we demonstrated that dapagliflozin attenuated cardiac hypertrophy and improved cardiac function.

### Dapagliflozin Down-Regulates the Levels of Genes Associated With Cardiac Hypertrophy in AAC-Induced Mice

**FIGURE 2:**
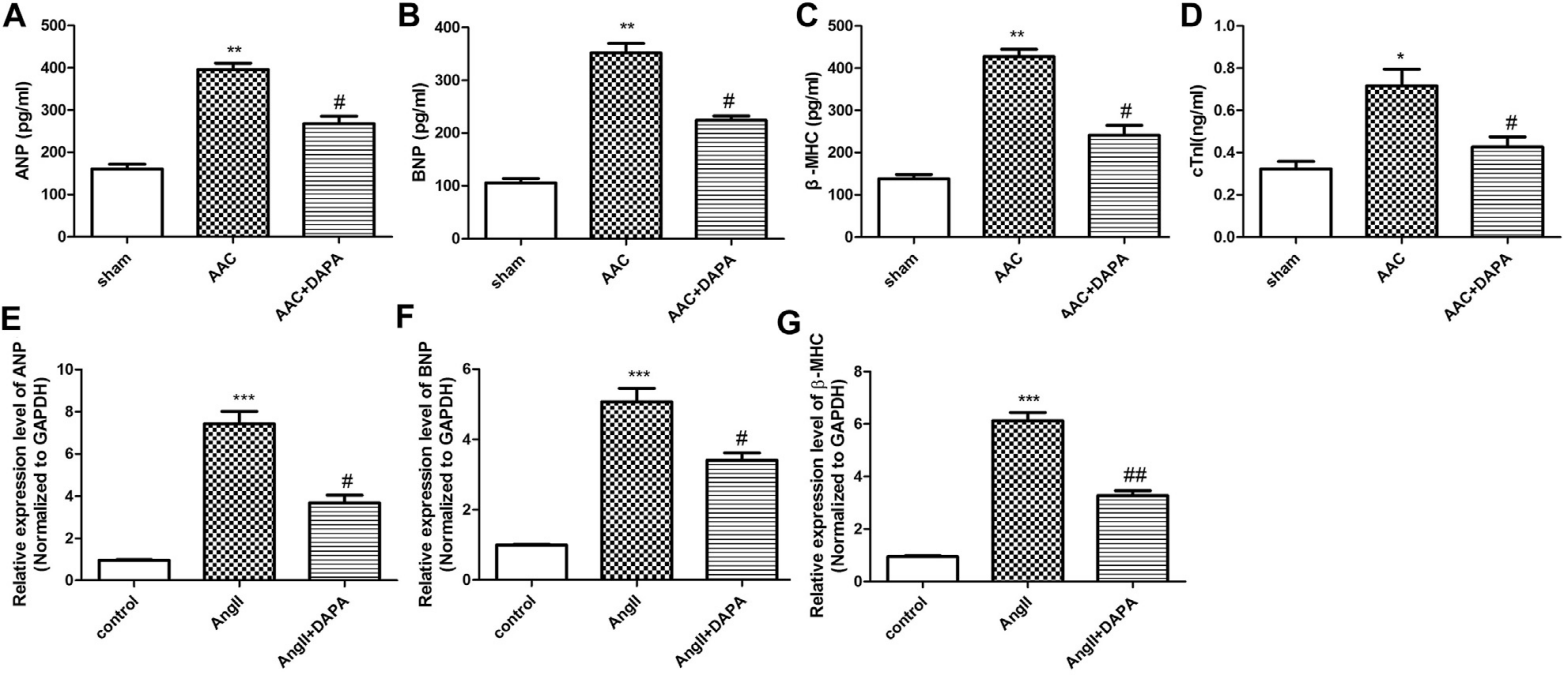
Effect of dapagliflozin on the level of cardiac hypertrophy-related genes in AAC-induced mice. The contents of ANP **(A)**, BNP **(B)**, β-MHC **(C)** and cTnI **(D)** in cardiac tissues of different groups were detected via ELISA kits. qRT-PCR analysis was performed to detect the mRNA expression of ANP **(E)**, BNP **(F)** and β-MHC **(G)**. The data were expressed as mean ± SD, *p < 0.05, vs. sham group; #*p* < 0.05, vs. AAC group.

In addition to the decreased cardiac function and the abnormal enlargement of the heart shape, cardiac hypertrophy is characterized by the activation of the fetal gene program, the abnormal up-regulated genes mainly including ANP, BNP, β-MHC and cTNI. In order to further confirm the effect of dapagliflozin on cardiac hypertrophy, ELISA assay was performed to detect the changes in the markers related to cardiac hypertrophy in serum of AAC-induced mice. As shown in Figures 2A–D, compared with sham group, the expression levels of ANP, BNP, β-MHC and cTNI in AAC group were significantly increased. After treatment, the expressions of markers were down-regulated by dapagliflozin. Moreover, qRT-PCR analysis was performed to detect the mRNA expression of ANP, BNP and β-MHC in heart tissues. Similarly, the qRT-PCR results (Figures 2E–G) were consistent with ELISA results. Based on the above results, we hypothesized that dapagliflozin could mitigate cardiac hypertrophy in AAC-induced mice.

### Dapagliflozin Attenuated AngII-Induced Cardiomyocyte Hypertrophy

**FIGURE 3:**
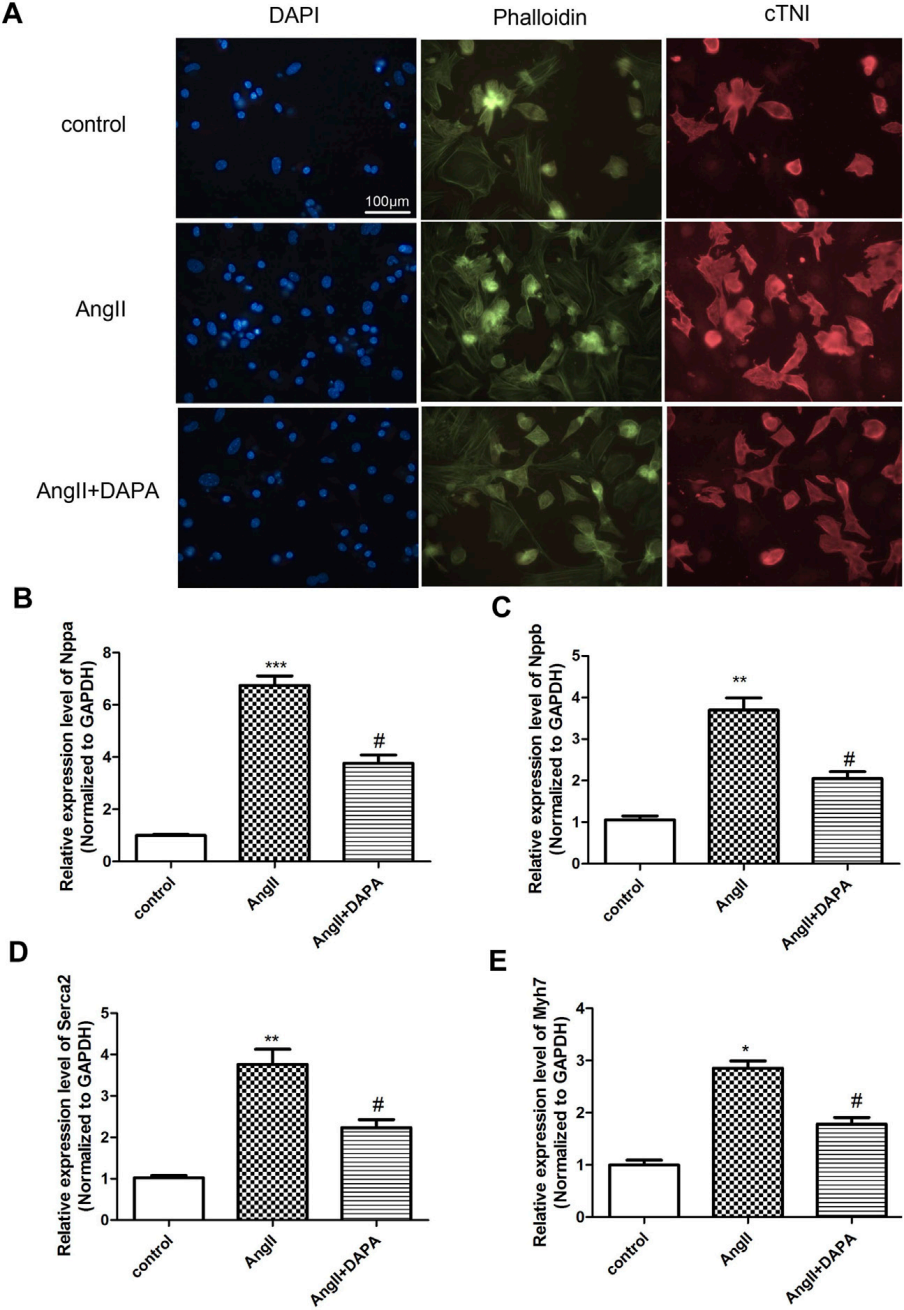
Dapagliflozin attenuated AngII-induced cardiomyocyte hypertrophy. **(A)** The morphological changes of cardiomyocyte cells were evaluated by immunofluorescence staining with anti-cTNI (red), cells were counterstained with DAPI (blue) staining the nucleus, Phalloidin-FITC staining the cytoskeleton (green) qRT-PCR analysis was used to detect the mRNA expression of Nppa **(B)**, Nppb **(C)**, Serca2 **(D)** and myh7 **(E)** in different groups. The data were expressed as mean ± SD in three independent experiments, *n* = 3, *p < 0.05, **p < 0.01, vs. control group; #*p* < 0.05, ##*p* < 0.01, vs. AngII group.

Stimulation of NMCMs by AngII could induce cardiac hypertrophy (Mele et al., 2019). Cardiac troponin I (cTnI) is an important structural protein and a cardiomyocyte-specific marker (Luo et al., 2021). Immunofluorescence staining results (Figure 3A) of NMCMs showed obvious hyperplasia of myocardial cells induced by AngII, suggesting that the cardiac hypertrophy model was successful. Cardiomyocyte hypertrophy was inhibited after dapagliflozin treatment, demonstrating that dapagliflozin attenuates cardiac hypertrophy *in vitro*. And, qRT-PCR analysis was performed to detect the expression level of genes related to cardiac hypertrophy and to evaluate the effect of dapagliflozin on cardiomyocyte hypertrophy *in vitro*. The results (Figures 3B–E) indicated that compared with control group, the mRNA expressions of Nppa, Nppb, Serca2 and myh7 in AngII group were up-regulated significantly. While the mRNA expressions of Nppa, Nppb, Serca2 and myh7 in treatment group was down-regulated markedly compared to AngII group. Taken together, the results indicated that dapagliflozin attenuated cardiomyocyte hypertrophy *in vitro*.

### Differential Gene Analysis of Dapagliflozin in AAC-Induced Mice Detected by Gene Microarray

**FIGURE 4:**
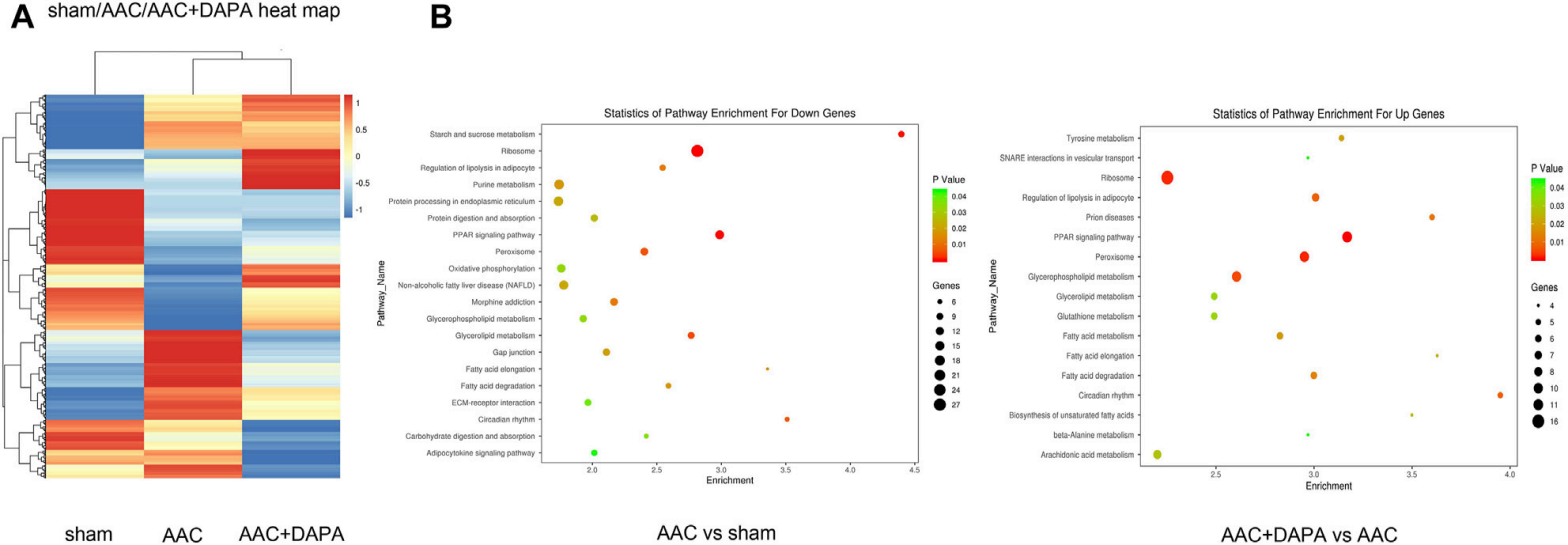
Differential gene analysis of dapagliflozin in AAC-induced mice detected by gene microarray **(A)** Clustered heat map showed the differential gene expression profile after dapagliflozin treatment in AAC-induced mice. **(B)** Signaling pathway enrichment analysis among different groups, the PPAR signal pathway (red line) is the signal pathway of this research.

To further study the protective mechanism of dapagliflozin in AAC-induced mice, we conducted a microarray analysis. FC > 1.5 or FC < −1.5 represented significant differential expression. As shown in Figure 4A, compared with AAC group, we found 2014 genes changed significantly in the dapagliflozin treatment group, among which 941 genes were up-regulated and 1,073 genes were down-regulated. Besides, signaling pathway enrichment analysis showed the PPAR signal pathway was significantly down-regulated in AAC-induced mice compared with sham group. While PPAR signaling pathway was up-regulated in treatment group compared with AAC group. In addition, we have identified some key differential genes (Table 1), which may be key signaling molecules for the protective effects of dapagliflozin. In subsequent experiments, we explored the mechanism of dapagliflozin by targeting signaling pathways and key molecules.

**TABLE 1:** Representative differential genes after dapagliflozin treatment in AAC-induced mice (Fc < 0, down-regulated; Fc > 0, up-regulated).

Gene name	Fold changes	
	Sham/AAC	AAC + DAPA/AAC
STAT1	3.227	−7.945
Plin5	−1.636	1.558
PDK4	−75.584	18.896
HMGCS2	−134.364	9.646
Cxcr4	2.014	−2.099
Tap1	2.056	−1.853
IGF1	1.879	−2.071
Sly	6.916	−8.754
Uty	188.706	−206.5
Rgs7	−4.199	1.753
Thy1	−1.705	1.705
Sdc2	−1.879	1.879
Vcp	−2.189	1.919
Gpx3	−2.173	2.099
Shb	−2.25	1.84

### Dapagliflozin Mediated the Plin5/PPARα Signaling Axis to Mitigate AngII-Induced Cardiomyocyte Hypertrophy

**FIGURE 5:**
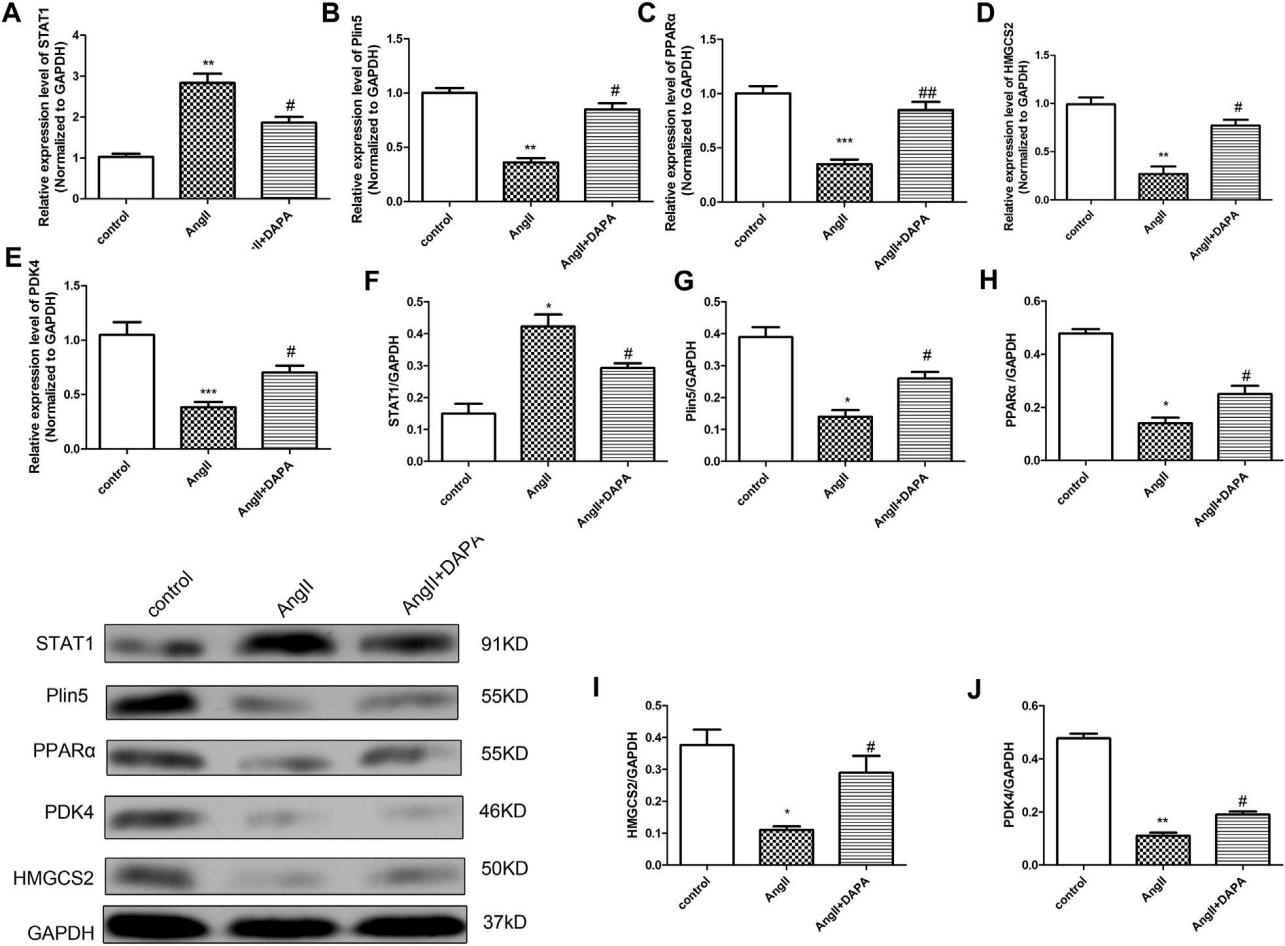
Dapagliflozin mediated the Plin5/PPARα signaling axis to mitigate AngII-induced cardiomyocyte hypertrophy. qRT-PCR analysis was used to detect the mRNA expressions of STAT1 **(A)**, Plin5 **(B)**, PPARα **(C)**, HMGCS2 **(D)** and PDK4 **(E)** in cardiomyocytes of different groups. **(F–J)** Western blotting assay was performed to detect the protein expressions of STAT1, Plin5, PPARα, HMGCS2 and PDK4. The data were expressed as mean ± SD, *n* = 3, *p < 0.05, vs. control group; #*p* < 0.05, vs. AngII group.

Combined with the differential genes screened by microarray, qRT-PCR analysis and western blotting assay were used for *in vitro* validation. We identified the differential gene Plin5, STAT1 and PPARα signaling pathways for verification. As shown in Figures 5A–E, qRT-PCR results showed that compared with control group, the mRNA expression of STAT1 was up-regulated significantly in AngII group, while the expressions of Plin5, PPARα, HMGCS2 and PDK4 were down-regulated. After treatment, the mRNA expressions of STAT1, Plin5, PPARα, HMGCS2 and PDK4 were reversed by dapagliflozin. Interestingly, the qRT-PCR results matched the microarray results. Likewise, western blot results (Figures 5F–K) showed the same trends. To sum up the above results, we hypothesized that dapagliflozin mediated the Plin5/PPARα signaling axis to mitigate cardiac hypertrophy.

### Dapagliflozin Mediated the Plin5/PPARα Signaling Axis to Attenuate Cardiac Hypertrophy *in vivo*

**FIGURE 6:**
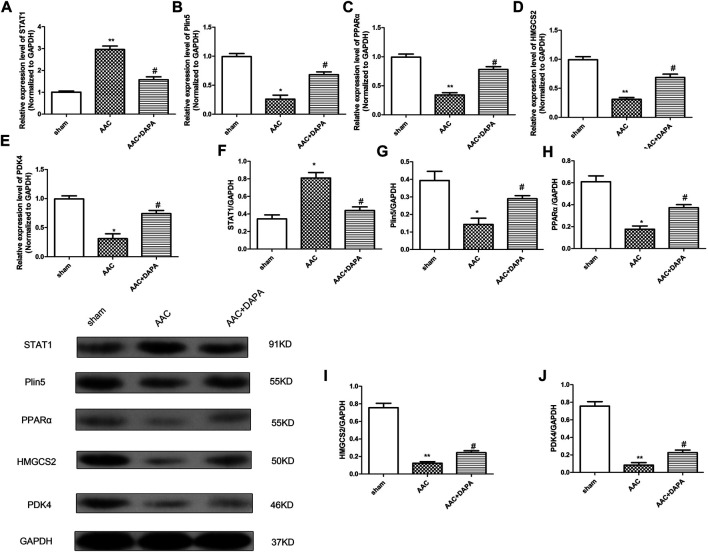
Dapagliflozin mediated the Plin5/PPARα signaling axis to attenuate cardiac hypertrophy *in vivo*. qRT-PCR analysis was used to detect the mRNA expressions of STAT1 **(A)**, Plin5 **(B)**, PPARα **(C)**, HMGCS2 **(D)** and PDK4 **(E)** in cardiac tissues. **(E)** Western blotting assay was performed to detect the protein expressions of STAT1, Plin5, PPARα, HMGCS2 and PDK4. The data were expressed as mean ± SD, *n* = 3, *p < 0.05, vs. sham group; #*p* < 0.05, vs. AAC group.

To further clarify our hypothesis, we validated the expression of differential genes and signaling molecules in AAC-induced mice. QRT-PCR analysis and western blotting assay were performed to detect the mRNA and protein expressions of STAT1, Plin5, PPARα, HMGCS2 and PDK4 in heart tissues of different groups. Similarly, we found the same results (Figure 6) as *in vitro* experiment, confirming that dapagliflozin could attenuate cardiac hypertrophy.

### Silence Plin5 or GW6471 Could Reverse the Protective Effect of Dapagliflozin-Mediated PPARα Signaling Axis in AngII-Induced Cardiomyocyte Hypertrophy

**FIGURE 7:**
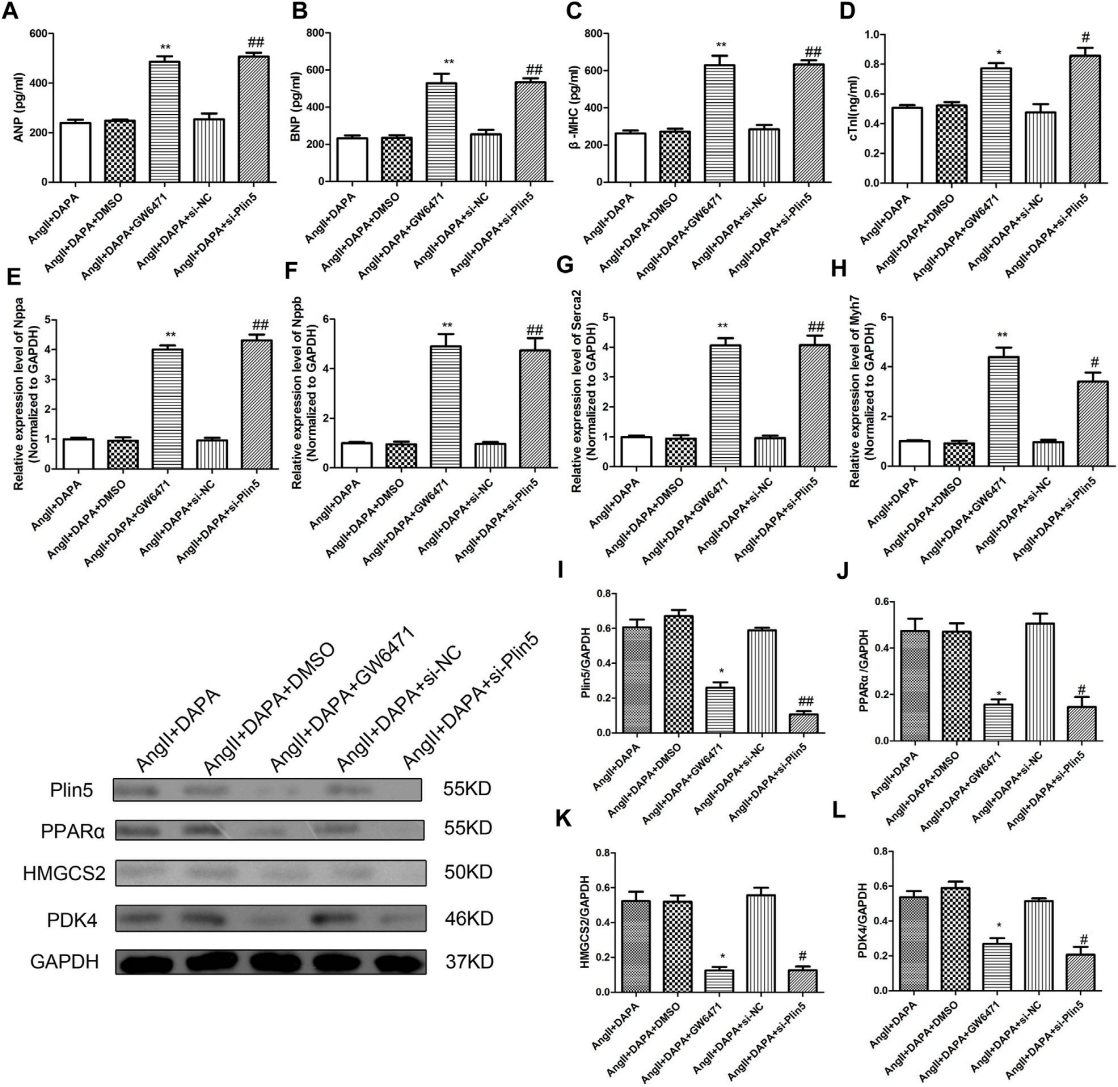
Silence Plin5 or GW6471 could reverse the protective effect of dapagliflozin-mediated PPARα signaling axis in AngII-induced cardiomyocyte hypertrophy. The content of ANP **(A)**, BNP **(B)**, β-MHC **(C)** and cTNI **(D)** in cell culture supernatant was detected by ELISA kits. qRT-PCR analysis was performed to detect the mRNA expression of Nppa **(E)**, Nppb **(F)**, Serca2 **(G)** and myh7 **(H)** in different groups. **(I–L)** Western blotting assay was used to detect the protein expressions of Plin5, PPARα, HMGCS2 and PDK4 in cardiomyocyte of different groups. The data were expressed as mean ± SD, *n* = 3, *p < 0.05, vs. AngII + DAPA + DMSO group; #*p* < 0.05, vs. AngII + DAPA + si-NC group.

Finally, we investigated whether dapagliflozin mediated the Plin5/PPARα signaling axis to exert a protective effect against cardiac hypertrophy. Plin5 silencing and GW6471 (PPARα inhibitors) were used to reduce the expression of Plin5 and PPARα, respectively. The expression levels of Plin5/PPARα signal axis-related proteins were detected by western blotting assay. The results (Figures 7A–D) showed that compared with AngII + DAPA + DMSO group, the expressions of Plin5, PPAR-α, HMGCS2 and PDK4 in AngII + DAPA + GW6471 group were reduced significantly. Similarly, the expressions of Plin5, PPARα, HMGCS2 and PDK4 in AngII + DAPA + si-Plin5 were significantly down-regulated compared with AngII + DAPA + si-NC group. Western blot results suggested that si-Plin5 and GW6471 could reverse the expression levels of Plin5/PPARα signaling axis in AngII-induced cardiomyocyte. Thus, we speculated that dapagliflozin could up-regulate the expressions of Plin5/PPARα and further promote the expression levels of PDK4 and HMGCS2. Subsequently, ELISA and qRT-PCR assays were used to detect the expressions of cardiac hypertrophy-related molecules and verify the effect of si-Plin5 and GW6471 on the protective effect of dapagliflozin. ELISA results (Figures 7E–H) showed that compare to AngII + DAPA + DMSO group, the contents of ANP, BNP, β-MHC and cTNI in AngII + DAPA + GW6471 group were significantly increased. The contents of ANP, BNP, β-MHC and cTNI in AngII + DAPA + si-Plin5 were increased markedly compared with that in AngII + DAPA + si-NC group. Furthermore, qRT-PCR results (Figures 7I–L) showed that compared to AngII + DAPA + DMSO group, the mRNA expressions of Nppa, Nppb, Serca2 and myh7 in AngII + DAPA + GW6471 group were significantly increased. The mRNA expressions of Nppa, Nppb, Serca2 and myh7 in AngII + DAPA + si-Plin5 group were increased significantly compared with those in AngII + DAPA + si-NC group. In conclusion, we found that si-Plin5 and GW6471 could reverse the protective effect of dapagliflozin on cardiac hypertrophy. It also revealed that dapagliflozin regulated the Plin5/PPAR-α signaling axis to inhibit cardiac hypertrophy.

## Discussion

In the present study, we evaluated the effect of DAPA on AAC-induced cardiac hypertrophy. *In vivo*, we found that DAPA treatment mitigated AAC-induced myocardial hypertrophy, fibrosis, and cardiac dysfunction. *In vitro*, we confirmed that DAPA inhibited AngII-induced abnormal cardiomyocytes hypertrophy. Furthermore, we demonstrated an inhibitory role of DAPA on cardiac hypertrophy by activating Plin5/PPARα signaling cascades in the myocardium. These results implied that DAPA could ameliorate cardiac dysfunction in AAC-induced mice, which is consistent with the results of other studies.

DAPA is a new class of oral hypoglycemic agents, SGLT2I, which can enhance renal glucose excretion or glycerine and reduce hyperglycemia (Garcia-Ropero et al., 2018). It has been reported that patients taking DAPA have a lower risk of heart failure and heart disease compared with other glucose-lowering drugs. There is sufficient evidence to suggest that the cardioprotective effects of DAPA are attributable to their systemic effects through glucose and sodium. However, the molecular mechanism of its regulation on cardiac hypertrophy remains unclear.

In the present study, AAC-induced cardiac hypertrophy model in mice was established to further investigate the cardiac function and pathological process after DAPA treatment. We found that DAPA significantly improved cardiac function and increased ejection fraction, which is consistent with Chang et al. reported results (Zhang et al., 2021). After 12 weeks of treatment, we found that the LVEF and LVFS were significantly increased, while LVEDV and LVESV were significantly decreased. In addition, we found that DAPA could reduce cardiac enlargement and inhibit the development of pathological processes, which may contribute to improving cardiac function.

Taking into account the complex pathological factors of cardiac hypertrophy, ventricular hypertrophy and left ventricular weight were considered as the core factors of cardiac hypertrophy aggravated death (Aistrup et al., 2013). In our study, DAPA treatment significantly improved the HW/BW ratio and inhibited myocardial tissue fibrosis. Besides, *in vitro* experiments also confirmed that DAPA can significantly reduce the abnormal hypertrophy of cardiomyocytes and downregulate the molecular markers related to cardiac hypertrophy.

PPARα is the most abundant PPAR isoform in heart tissue, and the activation of its related signaling pathways directly affected the expressions of key genes in fatty acid oxidation, which is crucial to lipid metabolism and energy metabolism balance in the heart (Campbell et al., 2002). Numerous studies (Zhao et al., 2017) have reported the beneficial effect of activation of the PPARα signal on the development of cardiac hypertrophy, which may be attributed to its regulation of myocardial function and energy metabolism through modulating fatty acid oxidation. Hrvey (Harvey et al., 2020) reported that Nox2 was a key signaling molecule in the pathological reaction of PPARα down-regulation leading to cardiac hypertrophy, which proved that the molecular mechanism of PPARα in cardiac hypertrophy from the negative side. In cardiac hypertrophy, the overexpression of PPARα in cardiac tissue can mediate the p53/GSK3β signaling pathway to significantly improve myocardial energy deficiency and cardiac function, which also supports the important role of PPARα in the pathological process of cardiac hypertrophy (Rana et al., 2019). Interestingly, the absence of PPARα led to a more marked hypertrophic growth response and cardiac dysfunction, which is associated with enhanced expression of inflammatory markers and extracellular matrix remodeling (Smeets et al., 2008). In our study, Plin5 and PPARα signaling pathways were selected as the key signaling molecules in AAC-induced mice according to gene chip detection. Consistent with previous studies (Hou et al., 2021), we found that PPARα was significantly down-regulated in AAC-induced mice. Myocardial hypertrophy was significantly inhibited after treatment, suggesting that DAPA may mediate the PPARα-related signaling axis. It has been reported (Zhao et al., 2019) that microRNA-370 mediated the Plin5-dependent PPAR signaling pathway to protect mice from myocardial ischemia/reperfusion injury. Wang et al. (2019) found that PLIN5 deficiency exacerbated cardiac hypertrophy by promoting oxidative stress.

We demonstrated that DAPA could activate the Plin5/PPARα signaling pathway and further significantly promoted the expressions PDK4 and HMGCS2 in cardiac hypertrophy. It has been reported (Palomer et al., 2009) that PPARs played key roles in the modulation of glucose homeostasis in cardiac cells by regulating PDK4 through PGC-1α. It has been reported (Thapa et al., 2018) that adropin treatment of cardiomyocytes can reduce the inhibition of PDH activity by regulating the expression of PDK4, thus affecting the metabolic balance of glucose in cardiomyocytes, indicating that PDK4 plays a key role in myocardial metabolism. Besides, HMGCS2 could combat metabolic abnormalities by transferring the flux of excess intramitochondrial acetyl-CoA. Upregulation of HMGCS2 can promote myocardial glucose oxidation (Singh et al., 2018). We suggested that DAPA mediated the Plin5/PPARα signal axis, and ultimately affected the expression level of PDK4 and HMGCS2, myocardial cell metabolism, and relieve the pathological progression of cardiac hypertrophy.

At present, dapagliflozin is mainly used in the first-line treatment of diabetes with remarkable effects (Bouter et al., 2020; Shah et al., 2021). Recent clinical studies not only focus on the pathological mechanism of diabetes but also involve the treatment of cardiovascular diseases (Cappetta et al., 2020; Nassif et al., 2021). In the study, we investigated the protective effect of dapagliflozin on AAC-induced cardiac hypertrophy and its mechanism of involving activation of the Plin5/PPARα signaling axis.

## Data Availability

The data presented in the study are deposited in the GEO repository, accession number GSE183120.
